# Inhibitory Effects of Ursolic Acid on the Stemness and Progression of Human Breast Cancer Cells by Modulating Argonaute-2

**DOI:** 10.3390/ijms24010366

**Published:** 2022-12-26

**Authors:** Wen-Ling Liao, Yu-Fan Liu, Tsung-Ho Ying, Jia-Ching Shieh, Yueh-Tzu Hung, Huei-Jane Lee, Chen-Yang Shen, Chun-Wen Cheng

**Affiliations:** 1 Graduate Institute of Integrated Medicine, China Medical University, Taichung 40433, Taiwan; 2 Center for Personalized Medicine, China Medical University Hospital, Taichung 40433, Taiwan; 3 Department of Biomedical Sciences, Chung Shan Medical University, Taichung 40201, Taiwan; 4 Department of Obstetrics and Gynecology, Chung Shan Medical University Hospital, Taichung 40201, Taiwan; 5 Institute of Medicine, Chung Shan Medical University, Taichung 40201, Taiwan; 6 Department of Biochemistry, School of Medicine, College of Medicine, Chung Shan Medical University, Taichung 40201, Taiwan; 7 Institute of Biomedical Sciences, Academia Sinica, Taipei 11529, Taiwan; 8 Graduate Institute of Environmental Science, China Medical University, Taichung 40433, Taiwan; 9 Department of Medical Research, Chung Shan Medical University Hospital, Taichung 40201, Taiwan

**Keywords:** breast cancer, ursolic acid, cancer stem cell, argonaute-2, PTEN

## Abstract

The stemness and metastasis of cancer cells are crucial features in determining cancer progression. Argonaute-2 (AGO2) overexpression was reported to be associated with microRNA (miRNA) biogenesis, supporting the self-renewal and differentiation characteristics of cancer stem cells (CSCs). Ursolic acid (UA), a triterpene compound, has multiple biological functions, including anticancer activity. In this study, we find that UA inhibits the proliferation of MDA-MB-231 and MCF-7 breast cancer cell lines using the CCK-8 assay. UA induced a significant decrease in the fraction of CSC in which it was examined by changes in the expression of stemness biomarkers, including the *Nanog* and *Oct4* genes. UA altered invasion and migration capacities by significant decreases in the levels of epithelial-to-mesenchymal transition (EMT) proteins of slug and vimentin. Furthermore, the co-reduction in oncogenic miRNA levels (*miR*-*9* and *miR*-*221*) was a result of the down-modulation in AGO2 in breast cancer cells in vitro. Mechanically, UA increases PTEN expression to inactivate the FAK/PI3K/Akt/mTOR signaling pathway and the decreased level of c-Myc in quantitative RT-PCR and Western blot imaging analyses. Our current understanding of the anticancer potential of UA in interrupting between EMT programming and the state of CSC suggests that UA can contribute to improvements in the clinical practice of breast cancer.

## 1. Introduction

The dissemination and colonization of distant lymph node organs in cancer cells that acquire genetic and/or epigenetic alterations in primary tumor cells are the primary causes of most cancer deaths [1,2]. Cancer stem cell (CSC) scenarios are supported by small side population (SP) cells, located within the tumor niche, and are responsible for maintaining the propagation of new tumor cells [3]. SP cells enable self-renewal, initiation, and differentiation in the cancer colony setting that has the advantage of highly invasive, metastatic, and drug-resistant potential of new tumor ones. The multidrug-resistant transporter protein, ATP-binding cassette superfamily G member 2 (ABCG2), has been implicated as enriched expression in CSC chemoresistance [4]. In our previous studies, in an attempt to isolate breast CSC cells (BCSCs), we separated tumor SP cells based on this transport gene with the high enzymatic activity phenotype responsible for the efflux of the fluorescent dye, Hoechst 33342, and then validated by examination of overexpression of stem cell transcription factors, including *Nanog*, *Oct4*, and *CD133*, which are reported as specific markers in representative CSCs [5,6]. Furthermore, CSCs with high levels of pluripotency are associated with oncogenic microRNA (miRNA) molecules, their aberrant expressions provided proliferative and repair capacities of DNA to increase resistance to apoptosis and allow them to survive in the face of anticancer treatment [7].

During recent decades, scientific research has made great efforts to identify deregulated RNA-binding proteins (RBPs) in cancer cell development. Argonaute-2 (AGO2), one of the members of the Argonaute family, is the crucial component of the RNA-induced silencing complex (RISC) with catalytic activity during small-RNA-guided gene silencing processes [8]. As a key regulator of miRNA biogenesis, AGO2 plays multiple roles in aspects of tumor cell tumorigenesis and progression, as well as has been reported to mediate tumor-promoting transcriptomic changes during carcinogenesis, including prostate, breast, gastrointestinal, ovarian, and endometrial cancers of human organs [9,10]. Mechanically, the AGO2-mediated RISC complex promotes cancer cell outgrowth, survival, and motility by suppressing the tumor suppressor PTEN (phosphatase and tensin homologue on chromosome 10) and activates phosphatidylinositol-3-kinase (PI3K)/AKT signaling [11]. The loss of PTEN is correlated with a human breast and prostate stem/progenitor subpopulation accompanied by mesenchymal characteristics with epithelial-mesenchymal transition (EMT) and macrometastasis [12,13].

Pentacyclic triterpenes (PT) are derived from five carbon isoprene units of medicinal herbs, fruits, and vegetables that are widely recognized to have a wide range of biological activities in the treatment of diseases, including diabetic complications [14,15], cardiovascular diseases [16], as well as potential immunomodulators to express anti-inflammatory effects [17,18]. One of the PT molecules, ursolic acid (UA, 3β-hydroxy-urs-12-en-28-oic acid), has been devoted to the antitumor characterization of in vitro and in vivo studies and clinical applications, supporting multifunctional bioactivities in cancer [19,20]. Among its numerous biological properties, in the present study, the antitumor effect of UA is of particular interest due to the diversity that interferes with BCSC biogenesis, shedding light on the pilot of UA for the restriction of AGO2-mediated miRNA expression in triple-negative breast cancer (TNBC). TNBC encompasses a subtype of breast cancers that lack the expression of the estrogen receptor (ER), the progesterone receptor (PR), and the human epidermal growth factor receptor 2 (HER2). Compared with other human breast cancer cell lines, TNBC MDA-MB-231 cells are known to have a higher proportion of CSC and have more invasive phenotypes [21]. Deregulation of stem cell-related signaling pathways, such as NF-kB, Wnt/β-catenin, Notch, and PI3K/p-Akt, lead to chemoresistance, recurrence, and metastasis in TNBC [22]. In this study, we explored the down-modulation of AGO2 in breast cancer treated with UA, which led us to understand the increase in the level of PTEN inversely associated with AGO2 expression in inhibiting breast cancer invasiveness. These findings are essential for the establishment of UA-permissive cancer treatment relevant to the novel role of the anticancer action of this PT compound in the down-modulation of AGO2 to improve the invasiveness of CSC-imposed tumor cells and the development of therapeutic approaches to improve future clinical cancer therapy.

## 2. Results

### 2.1. UA Inhibited Cell Proliferation in Breast Cancer Cell Lines

**Figure 1:**
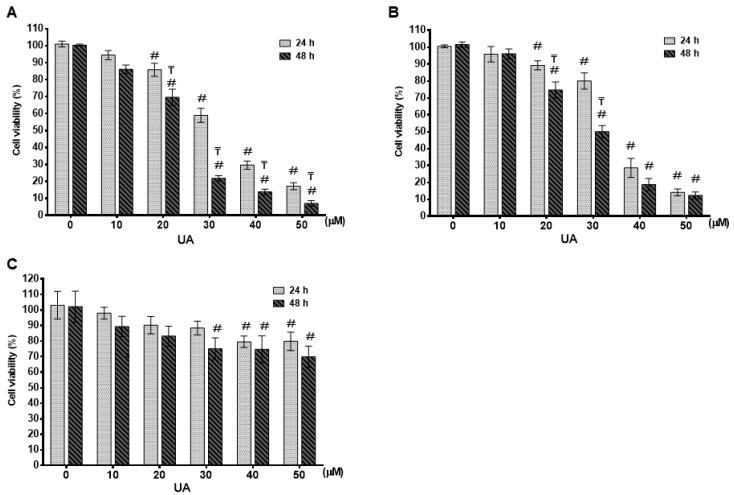
Effect of UA on cell survival in different breast cancer cells: Cell survival was evaluated by MTT assay after 24 h and 48 h of UA treatment in breast cancer cell lines of MDA-MB-231 in (**A**) and MCF-7 in (**B**) and normal mammary epithelial HBL-100 cell line in (**C**) with different concentrations as indicated. The control group (0 µM) did not receive UA treatment (100% cell viability). Data are shown as mean ± S.D for three independent experiments. #, indicates significant differences from the control; ₸, indicates significant differences compared to the respective concentrations; # and ₸ indicate significant differences at *p* < 0.05.

The effect of UA on the cell viability of different breast cell lines was evaluated using the CCK-8 assay. We used an achievably soluble dosing and tried several concentrations of UA for 24 and 48 h, respectively. UA caused a time (24 and 48 h)- and dosage (0–50 μM)-dependent inhibition of cell proliferation in MDA-MB-231 (Figure 1A) and MCF-7 (Figure 1B) cell lines (*p* < 0.001). At 24 h, the IC_50_ value of UA was greater than 30 μM for these two breast cancer cell lines. When incubation with UA was extended for 48 h, the IC_50_ values of UA on MDA-MB-231 and MCF-7 were calculated as 24.0 ± 1.8 μM and 29.2 ± 2.1 μM, respectively. On the contrary, as shown in Figure 1C, for HBL-100 human breast epithelial cells, treatment with more than 40 μM UA for 48 h decreased cell viability by less than 30% (74.7 ± 8.7% and 70.0 ± 6.8 with respect to 40 and 50 μM UA). These findings suggested that the MCF-7 and MDA-MB-231 carcinoma cell lines were more sensitive to UA treatment compared with the non-carcinoma cell line HBL-100. Furthermore, according to the status of the three most commonly used conventionally, namely ER, PR, and HER2, luminal A subtype MCF-7 and TNBC subtype MDA-MB-231 were selected for further study to investigate the inhibitory effects of UA on CSC, EMT and metastasis and to elucidate its role in the decrease in the signaling pathway with respect to the properties of CSC.

### 2.2. UA Decreases the Fraction of Breast Cancer Stem Cells

**Figure 2:**
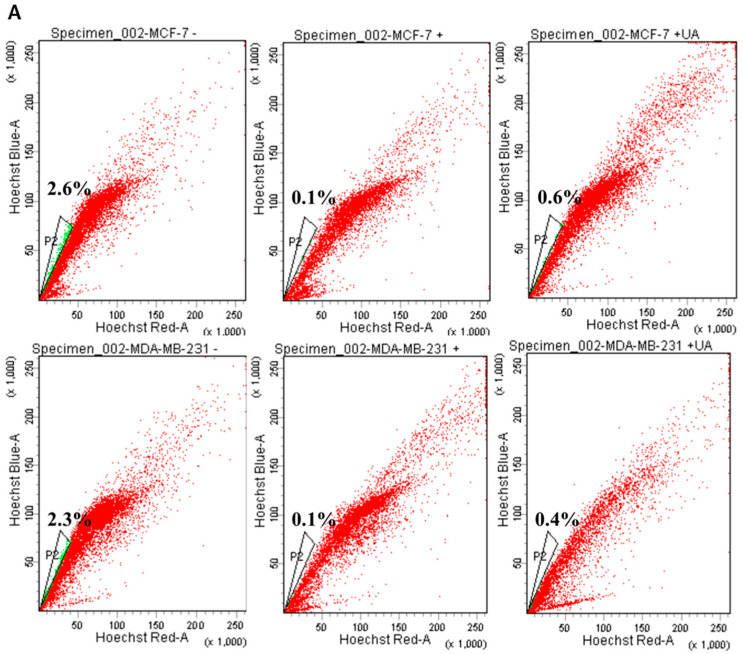

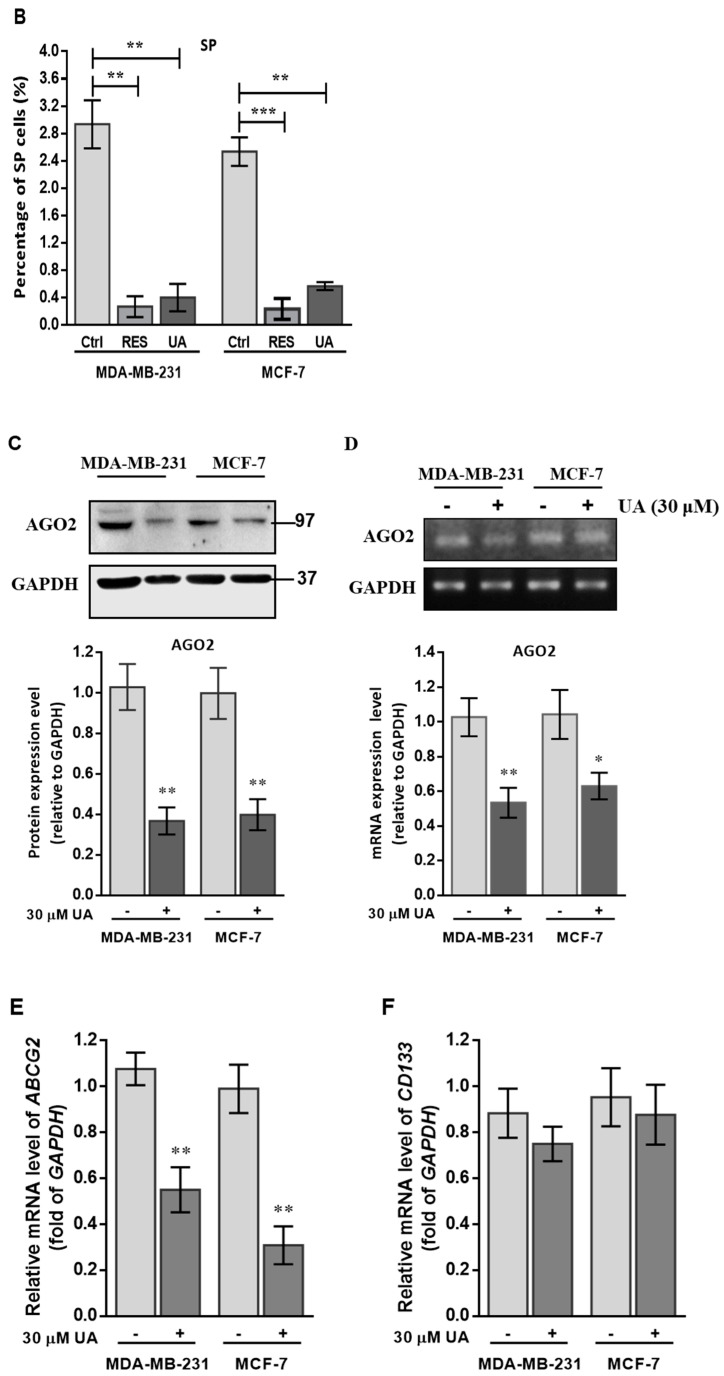

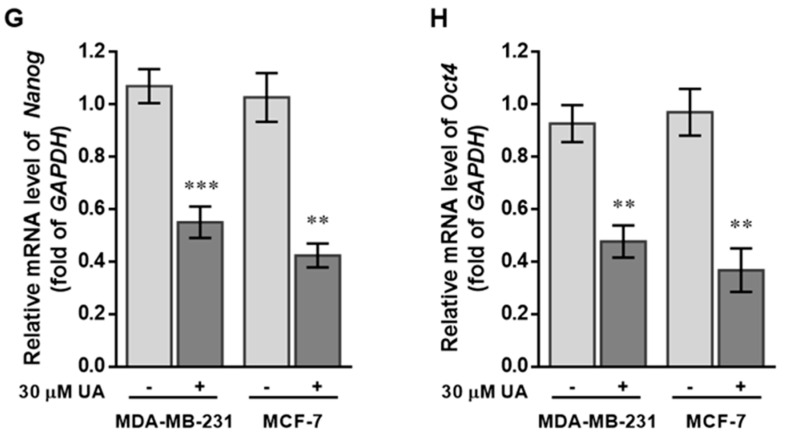
UA reduces the fraction of SP in breast cancer cell lines: (**A**) Representative examples of cancer stem cells (SP cells) were exploited by the flow-activated cell sorting (FACS) technique. Cells were stained with Hoechst 33342 dye in the absence (left) or presence (middle) of 50 µM reserpine and 30 μM UA (right) was evaluated by FACS. The uptake of Hoechst 33342 dye occurs uniformly in all cells through passive diffusion (shown in red), SP was detected by the loss of the fluorescent population (shown in green) after treatment with the ABCG2 inhibitor (RES) or UA. (**B**) The relative percentage of SP was estimated in human MDA-MB-231 and MCF-7 cell lines stained with Hoechst 33342 after incubation with UA for 48 h. (**C**) Western blot image and (**D**) reverse transcription-PCR (RT-PCR) data indicate that AGO2 levels in MDA-MB-231 and MCF-7 cell lines were significantly reduced after treatment with UA relative to control cells. (**E**) Reduced ABCG2 associated with lower levels of CSC-related genes encoding (**F**) *CD133*, (**G**) *Nanog*, and (**H**) *Oct4* in breast cancer cell line after UA treatment compared with the same cell line without UA treatment. GAPDH was used as an internal control for the detection of mRNA transcripts. Data were presented as mean ± SD from three independent experiments. * *p* < 0.05; ** *p* < 0.01; *** *p* < 0.001 vs. the untreated control.

In adult tissues or organs, the proportion of normal somatic stem cells is extremely rare. As previously stated, less than 0.01% (only an estimated 1 in 10,000 to 15,000) of hematopoietic cells are hematopoietic stem cells (HSC) in the HSC niche [23]. However, these induced pluripotent stem cells can be directed to CSC where an appropriate tumor microenvironment is present in the cancer niche and then lead to the fate of self-renewal and differentiation to perpetuate tumor regeneration of clonal evolution in cancer progression, and eventually developing drug resistance of the newly re-initiated cancer cell clone [24]. Therefore, we examine whether UA could influence the decreased proportion of CSC genesis of the MCF-7 and MDA-MB-231 breast cancer cell lines. Using Hoechst 33342 dye and fluorescence-activated cell sorting (FACS) of cancer cells treated with 20 µM reserpine, we concluded that after 48 h of incubation, UA significantly decreased the percentages of ABCG2-positive BCSCs, representing approximately 86% (from 2.3% to 0.4%) in MDA-MB-231 cells and 78% (from 2.6% to 0.6%) in the flow cytometry assay (Figure 2A,B). Furthermore, the significantly decreased level of AGO2 due to UA treatment in both breast cancer cell lines was estimated from Western blot (Figure 2C) and qRT-PCR analyzes (Figure 2D).

Increasing evidence has shown that CSCs are represented by SP cells associated with elevated levels of AGO2 and embryonic stem cell-specific factors, including the *ABCG2*, *Nanog*, *Oct4*, and *CD133* genes [25], we determined the relative transcripts of the genes in both cell lines, using real-time RT-PCR analysis and β-actin gene as internal standard for quantitative comparison (Figure 2E–H). Significantly decreased levels of mRNA transcripts were about 0.5-fold lower with respect to the *ABCG2* gene detected in both BC cells treated with UA compared to the control group (Figure 2E), as well as a significant reduction in CSC markers, including the *Nanog* and *Oct4* genes (by >60%) in cell lines treated with 30 μM UA compared to the control (Figure 2G,H). Together, these findings indicated that UA down-modulates AGO2 to restrict the renewal properties of CSC.

### 2.3. Sustained Exposure to UA Suppresses Migration and Invasion of Breast Cancer

**Figure 3:**
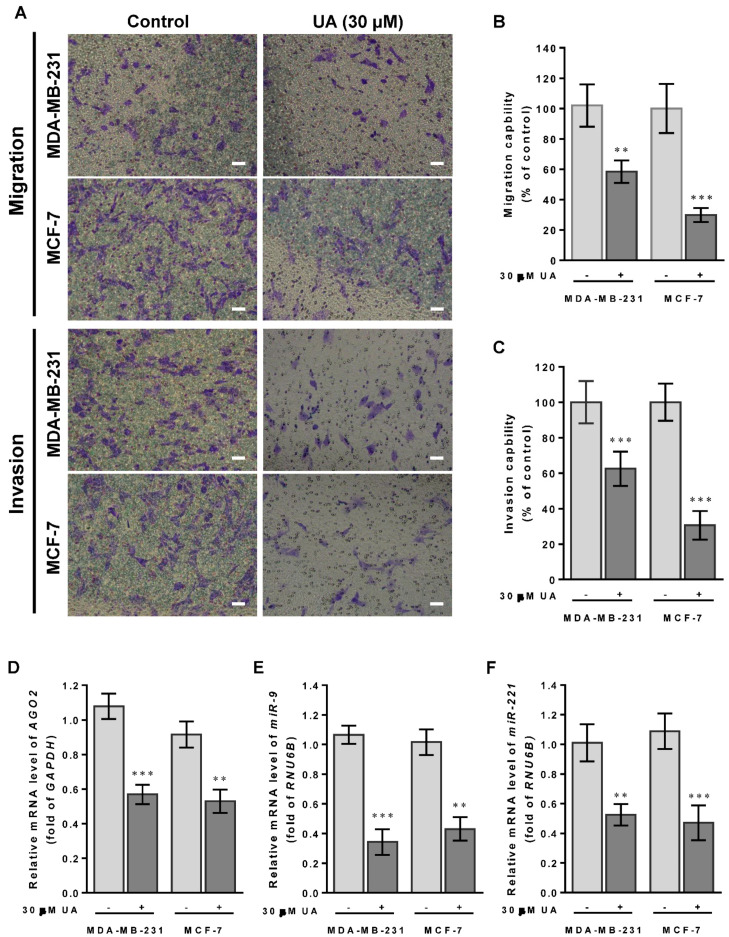

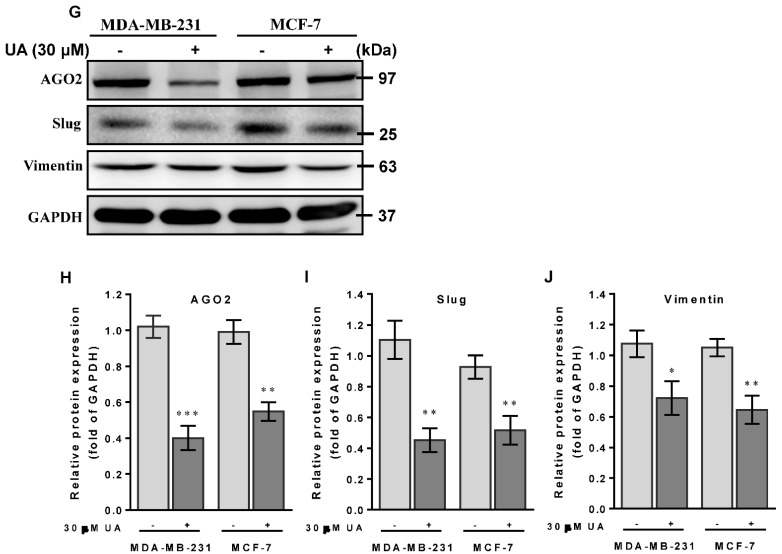
UA suppresses breast cancer cell migration and invasion: (**A**) Effects of UA on inhibition of cancer cell migration (top panel) and invasion (lower panel) for MDA-MB-231 and MCF-7 cells that were analyzed by the Transwell assay; scale bar = 50 μm. Quantification of migrated cells in (**B**) and invaded cells in (**C**) was presented as mean ± SD of three independent experiments. ** *p* < 0.01; *** *p* < 0.001 vs. untreated control. (**D**) Reduction in the *AGO2* gene and EMT-related miRNA in (**E**) *miR*-*9* and (**F**) *miR*-*221* in breast cancer cell lines treated with UA compared with the control. Relative expression of miRNAs is quantified using TaqMan-based real-time PCR along with *RNU6B*. (**G**) Representative images of Western blot of mesenchymal proteins and densitometric analyzes of AGO2 in (**H**), slug in (**I**), and vimentin in (**J**) were performed using Digital Protein DNA Imagineware. GAPDH was used as an internal control. All experiments were carried out in triplicate and all data are expressed as mean ± SD. * *p* < 0.05; ** *p* < 0.01; *** *p* < 0.001 vs. the untreated control group.

The CSC theory of cancer progression indicated that this subpopulation of cells plays a key role in driving tumor aggressiveness within the new tumor subclone, and therefore we examined the suppressive function of UA by reducing CSC to inhibit tumor cell metastasis of both breast cancer cell lines. As shown in Figure 3A, invasion and migration capabilities decreased significantly in both MDA-MB-231 and MCF-7 cells treated with UA for 48 h using the Transwell chambers assay. Compared with the control group, MDA-MB-231 cells treated with UA have significantly lower percentages of invasion and migration capacity by >40%. Similar results were obtained in MCF-7 cells treated with UA compared to those detected in the control group, showing a decrease in invasion and migration by >60% (Figure 3B,C).

### 2.4. UA Impedes Tumor Cell Migration and Invasion by Inhibiting EMT-Related miRNAs and Proteins

Furthermore, we have previously shown that *miR*-*9* and *miR*-*221* play an important role in contributing to the invasive phenotype due to the promotion of EMT in breast cancer [5]. Our hypothesis of the mechanism underlying the effects of UA on the decreased ability to invade and migrate casually to link *miR*-*9* and *miR*-*221* was due to the inhibition of AGO2 in breast cancer cells. In addition, because the MDA-MB-231 cell line is intrinsically deficient in E-cadherin expression [26] and the slug protein acts as a promoting transcription factor capable of increasing vimentin in invasive and metastatic cancers [27], we examined whether UA reduces slug and vimentin levels to prohibit migration and invasion of both breast cancer cell lines. As expected, we found a significant reduction in *miR-9* (Figure 3E) and *miR*-*221* transcripts (Figure 3F) when *AGO2* expression is repressed in the presence of UA treatment compared to the absence of UA in both breast cancer cell lines (Figure 3D). Given that decreased expression of these two miRNAs leads to EMT interruption and inhibits tumor metastasis, along with a reduction in AGO2 (Figure 3G), representative expression levels relevant to mesenchymal proteins, including slug and vimentin, were significantly lower in both breast cancer cell lines treated with UA than in negative control cells in Western blot image analysis (Figure 3H–J). Together, these data determined that UA treatment allows inhibition of breast CSC characteristics and the EMT process to eventually prevent breast cancer aggression.

### 2.5. UA Enhances PTEN to Downmodulate the FAK/PI3K/Akt/mTOR Signaling Pathway

**Figure 4:**
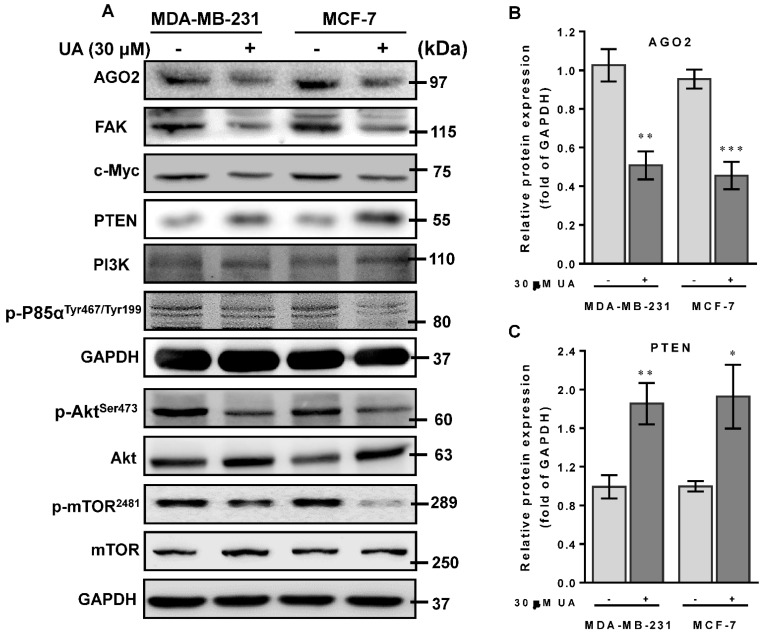

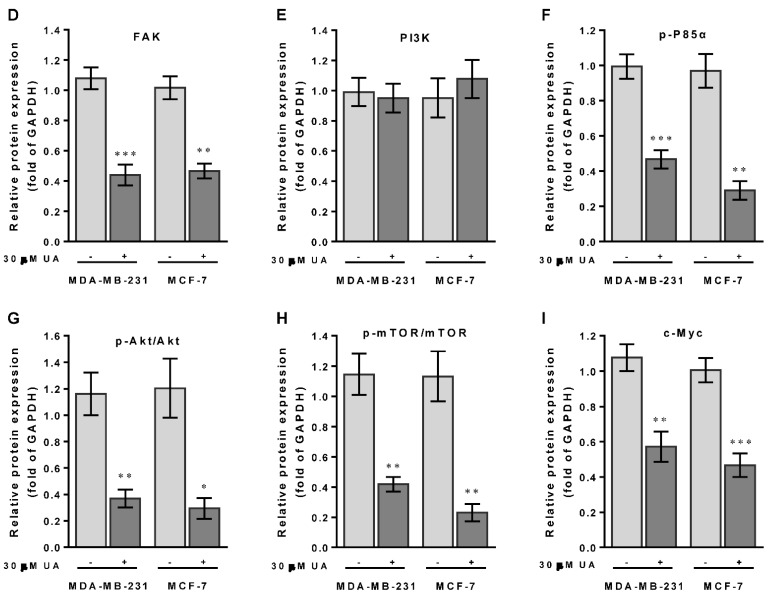
UA activates PTEN to modulate the FAK/PI3K/Akt signaling pathway: (**A**) Breast cancer cell lines were treated with UA for 48 h. Western blot analysis with antibodies to tumor progression-relevant proteins as indicated. GAPDH was used to normalize the amount of protein in each lane. (**B**) Incubation of breast cancer cells with UA (30 μM) significantly decreases AGO2. (**C**) Increased PTEN negatively regulates the expression levels of (**D**) FAK and (**E**) PI3-kinase p110 (PI3K), and inactivates the phosphorylated (activated) forms of (**F**) PI3K (p-P85α^Tyr467/Tyr199^), (**G**) Akt (p-Akt^Ser473^), and (**H**) mTOR (p-mTOR^2481^), and reduced level of (**I**) c-Myc in both cells treated with UA. Densitometric analyzes for Western blot were performed as described in Materials and Methods. Data are shown as mean ± SD for three independent experiments. * *p* < 0.05; ** *p* < 0.01; *** *p* < 0.001 vs. control group (Ctrl, 0 µM).

Recent studies have shown that PTEN gain of function could prevent ABCG2 activity with respect to tumor stem cell characteristics [28]. On the contrary, FAK acts as a key mediator in cell-ECM interactions that contributed to tumorigenesis and progression by stimulating AGO2 to activate the FAK/PI3K/Akt signaling pathway in head and neck squamous cell carcinomas [29]. Mechanically, we examined whether the reduced level of AGO2 due to UA could inactivate FAK/PI3K/Akt/mTOR signaling that retarded cell migration and invasion of breast cancer cells. As shown in Figure 4A, protein data from Western blot analysis showed that, in accordance with the reduction in AGO2 (Figure 4B), an increase in the level of PTEN protein (Figure 4C) was inversely associated with FAK expression levels (Figure 4D) and the phosphorylated status of PI3K (p-P85α^Tyr467/Tyr199^), Akt (p-Akt^Ser473^) and mTOR (p-mTOR^2481^) in the experimental group compared to those of MCF-7 and MA-MB-231 cells without UA treatment (Figure 4F–H). Furthermore, since AGO2 has been reported to stabilize c-Myc to facilitate stem cell renewal and differentiation, leading to tumorigenesis and progression of hepatocellular carcinomas [30]. Consistently, we also determined c-Myc expression levels that decreased along with AGO2 reduction after UA treatment (Figure 4I). These data support the underlying mechanism of UA that improves PTEN to decrease AGO2-mediated FAK/PI3K/Akt/mTOR signaling in conjunction with a reduction in c-Myc and pluripotent stem cell biomarkers, which is dedicated to the potential of UA as an effective natural phytochemical suppressor for breast cancer progression.

## 3. Discussion

In the present study, UA suppresses CSC and EMT to alleviate metastatic phenotypes by inhibiting AGO2 in breast cancer cell lines. As previously reported, AGO2 overexpression is crucial to turn on the transcription of numerous oncogenic proteins and RNA transcripts to promote pluripotent cell development and reprogram the tumorigenic potential of several types of cancer [31,32]. In addition, AGO2 overexpression contributes to the biogenesis of miRNAs that endorse biological activities in terms of self-renewal and differentiation of CSC [33,34]. In this paper, we explore that UA interferes with CSC characteristics through inhibition of AGO2; Downregulation of AGO2-mediated biogenesis of CSC-related miRNAs, such as *miR*-*9* and *miR*-*221*, by which the change in miRNA transcripts correlates with the reduction in slug and vimentin proteins during MET and prevents metastasis in MDA-MB-231 and MCF-7 breast cancer cell lines treated with UA.

UA has been shown to induce apoptosis and antimetastatic activity by destructing the PI3K/Akt/mTOR signaling pathways in human chronic myelogenous leukemia and breast cancer [35,36]. In this study, the metastatic potential of MDA-MB-231 and MCF-7 cells was significantly suppressed when both cell lines were treated with UA at a concentration of 30 μM. Mechanically, the effect of UA on the inhibition of breast cancer cell progression was determined by an elevated level of PTEN, as a consequence, the reduction in FAK associated with concomitant suppression of PI3K, Akt, and mTOR phosphorylation, leading to decreased capacity for invasion and migration of tumor cells. Interestingly, decreased levels of AGO2 and the phosphorylated form of m-TOR protein were detected in MDA-MB-231 cells treated with UA in which endocrine receptors, including ER, PR, and HER2, are deficient. This finding is consistent with the previous study showing that UA treatment inhibited phosphorylation of Akt and mTOR proteins in human LNCaP and PC-3 prostate cancer cells regardless of their status as androgen receptor or sensitivity to androgen [37]. Taken together, these data provide evidence to support the a priori theory that UA not only confers antitumor activities, but also prohibits an antimetastatic phenotype through suppression of AGO2 and inactivation of the FAK/PI3K/Akt pathway regardless of the status of the hormone receptor.

Recent studies have revealed that AGO2 acts as an essential mediator for the function of oncogenic miRNAs, such as *miR-19*, *miR-203*, and *miR-221/-222* which acquire the invasion and dissemination capabilities of mesenchymal stem cells [38,39,40]. Furthermore, the actions of *miR-9* and *-221* have been validated to correlate tumor clonal evolution and metastasis, manifesting an increase in the percentage of cells with CSC characteristics [5]. A distinguishing characteristic of neural stem cell (NSC) also showed increased levels of AGO2 associated with *miR-9* in the nucleus, leading to cell survival through the Notch signaling pathway of NSC cells [41]. In this study, cells treated with 30 μM UA demonstrated a significant reduction in *miR-9* and *-221* in SP cells of MDA-MB-231 and MCF-7. The molecular mechanism underlying the protection made by UA in antimetastatic properties is ascribed to a lesser extent to *miR-9*, shedding light on our understanding of miRNA-associated CSC genesis that correlates the expression level of the AGO2 protein. To our knowledge, the present study is the first of its kind investigation to demonstrate that UA impedes cancer cell stemming and tumor cell fates by inhibiting the AGO2-miRNA mechanism in human breast cancer.

**Figure 5:**
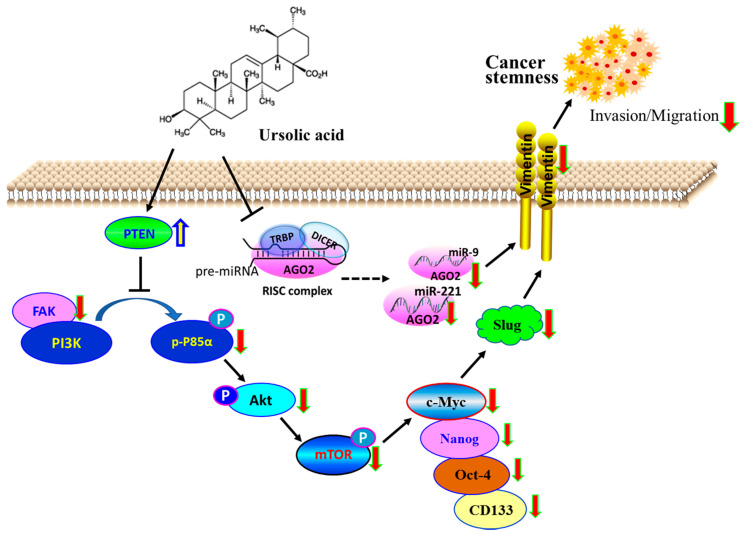
Schematic representation of anticancer activity through decreased BCSC. Tumor suppressive role of PTEN, whose gain in responding to UA decreases the fraction of CSC by downregulating the expression of AGO2 to inactivate the FAK/PI3K/Akt/mTOR and c-Myc signaling pathway. The model also suggests that UA decreased the AGO2-mediated biogenesis of *miR-9* and *-221* that contribute to the reduction in slug and vimentin, leading to the inhibition of breast cancer invasion and migration during EMT.

FAK acts as an important protein in cell–ECM interactions that affect cell proliferation, migration, and metastasis. Our results explored that inhibition of EMT-driven breast cancer cell metastasis by UA is endorsed by an increased level of PTEN in subsequent inactivation of the FAK/PI3K signaling pathway (Figure 5). Furthermore, AGO2 has been shown to decrease PTEN expression in HCC angiogenesis [42], whereas PTEN caused apoptotic death by repression of the c-Myc transcript in human breast cancer cells [43]. Consistently, we found that an increase in the level of PTEN is inversely associated with a decrease in the c-Myc transcript in MDA-MB-231 and MCF-7 cells treated with UA. The data confirmed that PTEN modulates the c-myc gene in which the significance of UA interferes with the malignant transformation of breast cancer. However, it should be noted that the broad spectrum of UA in anticancer activity is to interfere with proteins related to tumor cell characteristics of proliferation, adhesion, migration, and survival through numerous signaling pathways, including hypoxia-, PI3K/AKT-, Wnt/β-catenin signaling [44,45,46], as well as JAK2/STAT3 transcription in changing pro-tumor microenvironments [47]. In particular, when the effect of UA on breast cancer was examined, one major concern in evaluating this phytochemical in anticancer activity was that genetic contributions in relation to CSC characteristics and the current understanding of tumor cell pluripotency in breast cancer progression provides solid justification for our results.

## 4. Materials and Methods

### 4.1. Cell Culture

Two breast cancer cell lines, including invasive TNBC MDA-MB-231 (HTB-26) and early-stage MCF-7 [HTB-22, estrogen receptor positive, ER(+)] cell lines, and a non-tumorigenic human breast epithelial cell lines, HBL-100 (HTB-124) were obtained from the American Type Culture Collection (Manassas, VA, USA) and cultured in Dulbecco’s Modified Eagle’s Medium (DMEM, Life Technologies, Inc., Grand Island, NY, USA) containing 0.1 mM sodium pyruvate, 10% FBS, 2 mM L-glutamine, 100 IU/mL penicillin, 100 µg/mL streptomycin (Biosource, Rockvile, MD, USA) and supplemented with 10% fetal bovine serum. Cells were cultured at a constant temperature of 37 °C in a humidified atmosphere of 5% CO_2_.

### 4.2. Characterization and Isolation of CSC

Details for breast CSC preparation have been previously described [4]. Briefly, dissociated breast cancer cells (2 × 10^6^ cell/mL) were suspended in SP medium (calcium- and magnesium-free Hanks balanced salt solution with 2% FBS, 1% penicillin/streptomycin and 10 mM HEPES). Hoechst 33342 (Sigma-Aldrich, St. Louis, MO, USA) was optimized (5 μg/mL) on the respective breast tumor cells. The specific ABCG2 transporter inhibitor, reserpine (RES, Sigma-Aldrich) with a concentration of 50 μM was added before adding the Hoechst 33342 fluorescent dye. Cells were kept on ice in the dark for FACS analysis. The SP from NSP was then counted on a Becton Dickinson FACS Digital Vantage (Diva) cell sorter (BD Biosciences, San Jose, CA, USA). Hoechst was excited at a wavelength of 355 nm and a fluorescent profile was measured at dual-wavelength analysis (450/50 nm and 675/20 nm). Cells cotreated with Hoechst and RES were used as the control group for the determination of SP.

### 4.3. Cell Viability

The MTT [3-(4,5-dimethylthiazol-2-yl)-2,5-diphenyltetrazolium bromide] assay was performed exactly to estimate cell viability as previously described [48]. After UA treatment at the indicated times (24 and 48 h), cells (5 × 10^4^ cells/mL/well) were suspended and rinsed with phosphate-buffered saline (PBS) twice before incubation with MTT for an additional 4 h. The blue formazan generated, proportional to the number of viable cells in the MTT assay, was dissolved in isopropyl alcohol and measured spectrophotometrically at 563 nm. A percentage of cell viability with untreated cells was defined as 100%.

### 4.4. Matrigel Invasion and Migration Assays

The transwell cell migration assay that estimates the capacity for cell motility and invasiveness towards a chemo-attractant was performed exactly as previously stated [5]. Briefly, cells (5 × 10^4^) were seeded into 48-well modified Boyden chambers (Neuro Probe, Cabin John, MD, USA) with 8 μm pore size polyarbonate membrane filters to detect cell migration (without Matrigel) and invasion (containing Matrigel) for 24 h. Penetrated cells that were attached to the lower surface of the membrane were fixed with methanol and stained with Giemsa solution (Sigma-Aldrich, St. Louis, MO, USA). The invaded cells were stained with crystal violet dye and quantified by counting five random high-power visions using an Olympus Ckx41 light microscope (Tokyo, Japan).

### 4.5. RNA Extraction, cDNA Transcription and Real-Time PCR Analysis

Total RNA was extracted from breast cancer cells using TRIzol reagent (, Invitrogen Co., Carlsbad, CA, USA). First-strand cDNA synthesis was performed in the presence of random primers using a high-capacity cDNA reverse transcription kit (Applied Biosystems, Foster city, CA, USA) according to the manufacturer’s instructions. The sequences of the primer sets used for the real-time PCR analysis to detect each mRNA transcript are listed in Table 1. Real-time PCR was performed using the KAPA^TM^ SYBR^®^ FAST qPCR kit (KAPA Biosystems, Woburn, MA, USA) and the results were normalized to those of the glyceraldehyde-3-phophate dehydrogenase (*GAPDH*) gene. The sequences of probes and primer sets used to detect *miR-9* (ID 000583) and *miR-221* (ID 000524) in the microRNA assay were purchased from Applied Biosystems Inc. (Foster, CA, USA). The level of U6 small nuclear RNA (encoded by *RNU6B*) was used to normalize the relative expression level of the miRNAs. The comparative CT method (-ddCt) was used to estimate the relative expression (fold change) of mRNA and miRNA transcripts in tumor and non-tumor cells in each case (2^−ddCt^, where ddCt = dCt_target_ − dCt_internal control_). All qPCR experiments were performed in triplicate in the samples to determine the levels of target transcripts.

### 4.6. Western Blot Analysis

The preparation of whole cell extract and Western blot is performed as stated [5]. Protein concentrations were determined by BCA reagent following the manufacturer’s instructions (Bio-Rad, Richmond, CA, USA). All proteins were resolved on sodium dodecyl sulfate-polyacrylamide gels and transferred to PVDF membranes. Primary antibodies for AGO2 (no. 2897), phosphor-Akt (Ser473) (no. 9271S), phospho-mTOR (Ser2481) (no. 2974), slug (no. 9585) and vimentin (no. 5741) were purchased from Cell Signaling Technology (Danvers, MA, USA); ABCG2 (no. GTX12131), FAK (no. GTX100764), GAPDH (no. GTX100118) and PTEN (no. GTX101025) were purchased from GeneTex International Co., Ltd. (Hsinchu City 300 Taiwan.); phospho-PI3 kinase p85 (Tyr467/Tyr199)(# bs-3332R, Bioss, Inc., China); mTOR (# 66888-1, Proteintech) and PI3K (P110, #SC-8010) and c-Myc (#SC-40) were purchased from Santa Cruz Biotechnology (Santa Cruz, CA, USA). Horseradish-peroxidase-conjugated goat anti-mouse IgG antibody (Jackson Immunoresearch, West Grove, PA, USA) was used as the second antibody, and the signals were detected using the enhanced chemiluminescence (ECL) system (Millipore, Temecula, CA, USA). Protein band intensities quantified by densitometry (Digital Protein Imagineware, Huntington Station, NY, USA).

### 4.7. Statistical Analyses

All statistical analyzes were performed with GraphPad Pro Prism 8.01 (GraphPad, CA, USA). Continuous and categorical variables were displayed as mean ± standard deviation (SD) and percentages, respectively. The expression levels of the gene transcripts and the intensity of the Western blots of interest from the UA treatment were compared with the results from the control group using an unpaired two-tailed *t* test with Welch’s correction (Welch’s *t* test). The *p* value < 0.05 was considered statistically significant.

## 5. Conclusions

In conclusion, our findings provide evidence to support the tumor-suppressive role of PTEN, whose gain in responding to UA could decrease the CSC portion by downregulating the expression of AGO2. PTEN caused decreases in the levels of the FAK/PI3K/Akt/mTOR signaling pathway in association with EMT conversion in UA-treated breast cancer cells. Through this, UA has the ability to kill breast cancer cells, and animal studies are needed to prove this in vivo. Collectively, this may not only improve a signature of decreased cancer cell stemness that alters the communicative nature of EMT with respect to down-modulated AGO2 expression of UA, but also provide clinicians with information on new applications in alternative effective therapeutic routes for breast cancer.

## Figures and Tables

**Table 1:** Primer sets used for qRT-PCR analysis.

Gene	Primer Sequences Used for the mRNA Transcript		PCR Product
*AGO2*	Forward:	5′-TCCACCTAGACCCGACTTT-3′	155 bp
	Reverse:	5′-GTTCCACGATTTCCCTGTT-3′	
*ABCG2*	Forward:	5′-TCGGCTTGCAACAACTATG-3′	128 bp
	Reverse:	5′-TCCAGACACACCACGGATAA-3′	
*Nanog*	Forward:	5′-TAGCAATGGTGTGACGCAGAAG-3′	116 bp
	Reverse:	5′-TCTGGTTGCTCCACATTGGAAGG-3′	
*CD133*	Forward:	5′-ACAATTCACCAGCAACGAGTCC-3′	63 bp
	Reverse:	5′-GACGCTTTGGTATAGAGTGCTCAG-3′	
*Oct4*	Forward:	5′-GAGGCAACCTGGAGAATTTGTTCC-3′	64 bp
	Reverse:	5′-ATGTGGCTGATCTGCTGCAGTG-3′	
*GAPDH*	Forward:	5′-GAAGGTGAAGGTCGGAGTC-3′	226 bp
	Reverse:	5′-GAAGATGGTGATGGGATTTC-3′	

## Data Availability

Not applicable.

## Abbreviations

UA	ursolic acid
AGO2	Argonaute-2
CSC	cancer stem cell
SP	side population
ABCG2	ATP-binding cassette superfamily G member 2
EMT	epithelial-to-mesenchymal transition
miRNA	microRNA
PTEN	phosphatase and tensin homologue on chromosome 10
qRT-PCR	quantitative reverse transcription PCR
